# Leveraging HIV self‐testing to achieve the UNAIDS 2025 targets in the South and Southeast Asia region

**DOI:** 10.1002/jia2.26357

**Published:** 2024-10-13

**Authors:** Ishwarprasad S. Gilada, Michael M. Cassell, Chinmay S. Laxmeshwar, Davindren Tharmalingam, Vichea Ouk, Sathya Herath, Sovannarith Samreth, Nyan Oo, Rudi Wisaksana, Aryati Aryati, Rui Min Fung, Mauricio L. Nieto

**Affiliations:** ^1^ Unison Medicare & Research Centre Mumbai India; ^2^ HIV Department FHI 360 Durham North Carolina United States; ^3^ PATH Mumbai India; ^4^ Make It Right Movement (MIRM), BAC Education Group Petaling Jaya Malaysia; ^5^ National Center for HIV/AIDS, Dermatology and STD (NCHADS) Ministry of Health Phnom Penh Cambodia; ^6^ Unit of Key Population Programs & Program Implementation National STD/AIDS Control Program Colombo Sri Lanka; ^7^ Medical Department Save the Children International Yangon Myanmar; ^8^ Research Center for Care and Control of Infectious Diseases University of Padjadjaran Bandung Indonesia; ^9^ Department of Clinical Pathology Faculty of Medicine, Universitas Airlangga Surabaya Indonesia; ^10^ Vista Health Singapore Singapore

**Keywords:** HIV epidemiology, HIV testing, HIV self‐test, key and vulnerable populations, public health, South and Southeast Asia

## Abstract

**Introduction:**

The South and Southeast Asia region has the second‐highest number of people living with HIV globally. Despite progress in reducing HIV incidence and AIDS‐related deaths, the region still has a long way to go in achieving the Joint United Nations Programme on HIV and AIDS (UNAIDS) 95‐95‐95 HIV testing, treatment and viral suppression targets. HIV self‐testing (HIVST) is recommended by the World Health Organization as an additional approach to HIV testing. This paper provides a commentary on the implementation status, benefits, barriers and recommendations for HIVST implementation in South and Southeast Asia. Additionally, it presents perspectives from HIV testing service experts from 11 countries in the region to put forth recommendations to accelerate the implementation of HIVST in South and Southeast Asia.

**Discussion:**

There is uneven progress in national HIVST policy development and implementation across the region. HIVST, as an additional testing approach, can help to enhance testing coverage, frequency and demand for follow‐up HIV services among key populations. Key factors influencing the implementation and scale‐up of HIVST include the degree of awareness of HIVST among general and key populations, the development and implementation of supportive national HIVST policies and the availability of public funding for HIVST. To address barriers and leverage enablers to HIVST implementation, generating evidence on cost‐effectiveness and budget impact, developing multisectoral partnerships for market shaping, promoting differentiated and decentralized delivery models, and optimizing linkage to further testing and care are recommended.

**Conclusions:**

It is crucial to accelerate the implementation and scale‐up of HIVST to differentiate and decentralize the delivery of HIV testing services in South and Southeast Asian countries. Sharing experiences among country experts is vital to foster the adoption of best practices and facilitate the trial‐and‐error process of HIVST implementation. Such collaborative approaches can help South and Southeast Asian countries attain the UNAIDS 95‐95‐95 targets, especially the first 95 on HIV diagnosis, and play a significant role in ending the global AIDS epidemic.

## 1 INTRODUCTION

South and Southeast Asia is home to 3.5 million people living with HIV, making it the second most impacted region globally [1, 2]. HIV stigma and discrimination remain prevalent, discouraging both the general population and disproportionately affected key populations from accessing testing and care services. Progress in the region to achieve the Joint United Nations Programme on HIV and AIDS (UNAIDS) 95‐95‐95 testing and treatment targets to end the AIDS epidemic remains insufficient, with the most significant gap being the first target to have 95% of people living with HIV know their HIV status. As of 2022, progress in achieving these targets in Asia‐Pacific fell short at 78‐84‐95 [3]. Furthermore, there is uneven progress in the response to the AIDS epidemic among countries across the Asia‐Pacific region [4].

HIV testing serves as the gateway to treatment, management and prevention [5]. However, most South and Southeast Asian countries continue to rely on a narrow range of HIV testing approaches deployed primarily in health facilities and laboratory settings or conducted by trained lay providers in the community [6]. While the region has made noteworthy advancements in enabling access to HIV testing through centralized and facility‐based approaches, there remain challenges regarding geographical access barriers, limited testing capacity, lack of trained providers, and insufficient funding for central laboratory and facility‐based testing services.

The World Health Organization (WHO) strongly recommends offering HIV self‐testing (HIVST) as an additional approach to HIV testing [7]. HIVST is the process of collecting one's own specimen (oral fluid or blood) using an HIV self‐test kit, performing the test and interpreting the results [8]. HIVST allows users to test according to their own preferences and needs.

Multiple studies in South and Southeast Asia have associated HIVST with high acceptability and uptake among key populations, given its ability to offer confidentiality, privacy and convenience compared to centralized and facility‐based testing, helping to address barriers associated with stigma and discrimination [9, 10, 11, 12, 13]. Therefore, HIVST can help to increase the uptake and frequency of HIV testing, including among key populations often missed by existing HIV testing services [7, 8]. A randomized trial among men who have sex with men and transgender women in Myanmar reported that approximately 90% assigned to facility‐based HIV testing services would test more frequently if they had access to HIVST [13]. The *HIV Awal/Early Testing and Treatment Indonesia* study found that the implementation of HIVST atop facility‐based testing increased the average number of men who have sex with men who tested for HIV each month by 52% [14]. In Thailand, a recent study among key populations who had never accessed HIV testing services found that when presented with the choice of HIVST or referrals to facility‐based testing, the majority (72%) preferred HIVST [10].

HIVST uptake and reach among hard‐to‐reach populations can be further bolstered through innovative community‐based and virtual delivery strategies designed for key populations [15]. Secondary distribution models, where individuals (referred to as indexes) distribute HIVST kits to people within their social networks (referred to as alters), have been shown to be effective in reaching first‐time testers, as demonstrated in India's STAR III Initiative where 68.6% of participants reached by secondary distribution reported to be first‐time testers [16].

Blood‐ and oral‐based HIVST kits have demonstrated high clinical performance and usability, making them suitable for lay users to use safely, reliably and accurately [7, 8]. A study among Vietnamese lay users without prior experience or training in self‐testing found that blood‐based HIVST kits accurately identified 96.4% of HIV‐positive samples (sensitivity) and 99.9% of HIV‐negative samples (specificity) [11]. These kits have, therefore, been increasingly utilized in research and programs across low‐ and middle‐income countries as an additional approach for assay 0 (A0) test for triage [14, 16, 17]. While both blood‐ and oral‐based HIVST are recognized as highly accurate, blood‐based HIVST has been perceived by users to be more accurate [18, 19]. This perception is supported by existing performance evidence, which shows slightly higher sensitivity and specificity for blood‐based HIVST [20]. On the other hand, users typically perceive oral‐based HIVST as easier to use [18, 19]. This highlights the need to integrate various types of HIVST kits into the broader HIV testing continuum to cater to the diverse needs and preferences of different individuals.

This paper provides a commentary on the implementation status, evidenced benefits, barriers and enablers of HIVST in South and Southeast Asia and discusses the way forward to leap over the last mile of the UNAIDS 95‐95‐95 targets in the region. In addition to relevant evidence sourced from the literature, this commentary presents perspectives among HIV testing service experts, some of whom are co‐authors of this paper. Experts representing 11 South and Southeast Asian countries—Cambodia, India, Indonesia, Lao PDR, Malaysia, Myanmar, Pakistan, Philippines, Sri Lanka, Thailand and Vietnam—contributed to an expert discussion session on recommendations to accelerate the implementation of HIVST in the region. Additionally, a global expert and co‐author, as well as experts from Taiwan and South Africa, supplemented the discussions by providing perspectives and key learnings beyond South and Southeast Asia. Experts' profiles comprised government representatives (e.g. senior management within local Ministries of Health), community and development partners (e.g. leadership within national and international HIV and AIDS programs such as PATH) and technical experts (e.g. academics and clinical researchers specializing in HIV and AIDS).

## 2 DISCUSSION

### 2.1 National HIVST policy status

South and Southeast Asian countries continue to face challenges in addressing HIV diagnosis gaps. The lack of differentiation in testing approaches and the absence of supportive policies to expand HIVST access among key populations limit the reach of national HIV testing services. Experts representing 11 South and Southeast Asian countries discussed the status of national health policies favouring the use of HIVST in their respective countries, classifying these policies into one of three discrete stages: “No HIVST policy” (countries lacking guidelines, regulations or frameworks related to HIVST), “HIVST policy adopted” (countries where relevant authorities have officially endorsed or approved HIVST policies) and “HIVST policy implemented” (countries where HIVST policies have been put into action through the rollout of programs, services and initiatives on a large scale).

As of August 2022, India, Indonesia and Pakistan lacked national HIV testing policies recommending the use of HIVST. Lao PDR, Malaysia and the Philippines showed significant progress and were at the stage of adopting a national HIVST policy. Notably, Cambodia, Sri Lanka, Thailand and Vietnam had reached the stage where HIV policies enabled the rollout of HIVST programs. Progress in implementing national HIVST policies is uneven among the countries in the region. Beyond development, effective adoption and implementation of HIVST policies are crucial in enabling a differentiated range of centralized and decentralized testing approaches to provide key populations with options that cater to their preferences and to reach underserved and hard‐to‐reach populations.

### 2.2 Barriers and enablers to implementing HIVST

In line with WHO's guide for planning, introducing and scaling up HIVST [8], experts identified and assessed the top barriers and enablers to HIVST implementation from the perspective of their respective countries. The greatest barriers to implementing and scaling up HIVST include the lack of awareness about HIVST, limited government funding, absence of national HIVST policy, and inadequate supply and quality of HIVST kits. Conversely, key enablers to implementing and scaling up HIVST are the increasing acceptability and awareness of HIVST, progress in national HIVST policy implementation and government funding availability.

Lack of awareness of HIVST remains a challenge in some South and Southeast Asian countries, particularly among vulnerable populations such as adolescents at risk of acquiring HIV, older adults with lower education levels and individuals facing discriminatory attitudes [15, 19, 21, 22, 23]. WHO highlights the importance of focused HIVST demand generation and support tools to create awareness among key populations while preventing unnecessary retesting in groups at low risk of acquiring HIV [8]. The use of digital platforms like messaging apps, social media and partner‐seeking sites can help to increase reach, connect with key populations and reassure them of potential hesitancies towards HIVST [8, 15]. In the current digital era, men who have sex with men, transgender women and female sex workers are increasingly moving to virtual platforms, rendering them hard to reach and inaccessible [15].

Although WHO has recommended the adoption of HIVST as an additional testing approach since 2016, most South and Southeast Asian countries are still in the process of developing or adopting national HIVST policies. Progress has been uneven in the region, with only a few countries, including Cambodia, Sri Lanka, Thailand and Vietnam, achieving policy implementation. Overcoming scepticism among governments and practitioners and addressing regulatory barriers to HIVST kit registration are crucial steps to establishing HIVST as a standard public health practice [10].

Government funding plays a significant role in HIVST program sustainability and user affordability, which is a crucial factor influencing the decision of key populations to self‐test. Many users expect HIVST services to be covered by the government or other funders, leading to a preference for partially or fully subsidized HIVST kits [12, 24]. Monitoring users' willingness to pay across different sub‐populations and regions is important for identifying optimal pricing strategies. For instance, among Thai men who have sex with men and transgender women, HIVST kits priced between USD 7.70 and USD 9.30 were perceived as affordable and reliable, while prices above USD 10.00 would require subsidization to ensure affordability [10]. Exploring public‐private partnerships, cost‐control initiatives and insurance coverage can help foster sustainable national HIVST programs.

### 2.3 Recommendations to accelerate the implementation of HIVST in South and Southeast Asia

The following four recommendations have been discussed among experts in the region to help address the aforementioned barriers and capitalize on enablers of HIVST implementation to deliver broader public health benefits in South and Southeast Asia.

Provide preferred and differentiated HIVST delivery models: While HIVST allows for diverse access pathways to HIV testing, it is important to differentiate the delivery models and test kit options. A universal HIVST delivery model cannot effectively address the region's diverse geographical and infrastructural challenges nor cater to the varied user preferences and willingness to pay. India's STAR III Initiative demonstrated the importance of providing users with options that align with their preferences in their self‐testing journey. This approach has reached a remarkable acceptance rate reported at 96% among key populations [16]. Furthermore, digital approaches and home delivery options have the potential to reach individuals in remote areas. These approaches help address stigma, ensure privacy and foster stronger community engagement. Programs must prioritize monitoring the preferences of key populations to develop optimal HIVST access strategies that cater to diverse populations.

Generate evidence to shape policy and guide implementation: To accelerate the development of supportive policies and effective HIVST delivery models, expanding the evidence base in three key areas is crucial. First, there should be evidence of the cost‐effectiveness and budget impact of HIVST in South and Southeast Asian countries. Such evidence and evaluations are currently lacking in the region but are necessary to support policy, funding decisions and sustainable allocation of domestic health budgets and donor funds. Second, evidence assessing the impact of HIVST delivery models on generating demand for follow‐up HIV services, subsequent testing and care. For example, by complementing existing HIV testing strategies for pre‐exposure prophylaxis, HIVST can potentially increase both pre‐exposure prophylaxis use and HIV testing frequency [25]. Finally, further research can help corroborate the current evidence pointing to the minimal risks associated with HIVST in terms of gender‐based violence and negative social consequences. This, in turn, can mitigate unfounded hesitations and misconceptions.

Develop multisectoral and public‐private partnerships: Scaling up HIVST requires multi‐sectorial collaboration beyond just the health sector and partnerships between public and private sectors. Additionally, involving communities in the design of HIVST demand generation and delivery models deepens accurate understandings of market conditions while fostering community ownership. Engaging community‐based organizations in policy design ensures that guidelines are grounded in the needs and experiences of key populations. Private stakeholders and development partners can play a supportive role in optimizing the accessibility and availability of test kits. By providing initial funding for piloting HIVST programs, local evidence can be generated to support government reimbursement decisions later on, facilitating the transition to domestic funding for HIVST. Full or partial subsidy models can be implemented to support the sustainability of programs aiming to scale up HIVST nationwide. Collaboration with media outlets is essential in raising awareness about HIVST and ensuring that accurate and tactful messages are conveyed for promotional, recruitment and educational purposes. Finally, international technical agencies such as the WHO and UNAIDS should continue providing guidance to countries less experienced with HIVST.

Optimize linkage to further testing and care: Strategies to improve the linkage between HIVST and prevention services, confirmatory testing and care should uphold the convenience and privacy offered by HIVST [26]. In upholding the promise of privacy offered by HIVST, reporting should be voluntary and not a prerequisite for linkage to care. To illustrate, an HIVST program in Taiwan explored online self‐reporting options coupled with discounted HIV treatment at designated hospitals to encourage subsequent testing and care. This approach resulted in 49% of users voluntarily self‐reporting their test results online, and 60% of those with a positive result obtained a confirmatory diagnosis within a month [27]. This demonstrates the potential of HIVST in achieving linkage to subsequent testing, especially when strategically delivered with behavioural strategies (e.g. using financial incentives as a motivator).

There are some limitations to this commentary. As it builds upon the perspectives of experts from 11 countries in South and Southeast Asia, it may not be generalizable to other countries within the region. Insights from global, Taiwan and South Africa experts, as well as published literature, suggest similar barriers to HIVST implementation, such as low awareness, limited government policy and funding, and inadequate HIVST supply and quality. While the barriers to HIVST implementation may vary in magnitude across regions, lessons learned and best practices can still be adapted, localized and contextualized for application in other settings.

## 3 CONCLUSIONS

The need to accelerate the implementation of differentiated HIV testing strategies is underscored by the urgency of diagnosing people living with HIV to open the gateway to care and the prevention of HIV acquisition among key populations. HIVST, complementary to existing HIV testing services, shows promise in decentralizing testing approaches to expand testing coverage among key populations. To make HIVST an integral component of HIV testing services, government support and public‐private partnerships are crucial to achieving the implementation of national HIVST policies, sustainable domestic funding and availability of quality‐assured HIVST kits.

Experts in South and Southeast Asia can leverage shared experiences across countries by disseminating best practices and successful cases to facilitate the trial‐and‐error process of HIVST implementation and scale‐up. By doing so, countries in South and Southeast Asia can have more opportunities to progress and leap over the last mile of the UNAIDS 95‐95‐95 targets. As emphasized by UNAIDS in its 2022 Global AIDS Update, “If there is one lesson that the COVID‐19 pandemic has taught us, it is that pandemics can't be ended anywhere until they are ended everywhere” [28].

## COMPETING INTERESTS

CSL and SH received travel support from Abbott Rapid Diagnostics to attend the expert discussion session in Ho Chi Minh City, Vietnam. ISG received travel support and honorarium from Abbott Rapid Diagnostics to attend the expert discussion session meeting in Ho Chi Minh City, Vietnam. The remaining authors in this paper declare that they have no known competing financial interests or personal relationships that could have appeared to influence the work reported in this paper.

## AUTHORS’ CONTRIBUTIONS

ISG, MMC, CSL, DT, VO, SH, SS, NO, RW and AA contributed to insights generation. MLN and RMF developed the paper outline and initial draft. ISG, MMC, CSL and DT refined the initial and subsequent drafts. All authors reviewed the full paper.

## FUNDING

Abbott Rapid Diagnostics is the sponsor of the expert discussion session and this paper.

## DISCLAIMER

RMF and MLN are employees of Vista Health, an organization compensated by Abbott Rapid Diagnostics for the development of this paper.

## Data Availability

The author has provided the required Data Availability Statement, and if applicable, included functional and accurate links to said data therein.

## References

[1] The Foundation for AIDS Research . Funding gaps could put Asia further behind on HIV/AIDS. The Foundation for AIDS Research; 2016.

[2] Wang H , Wolock TM , Carter A , Nguyen G , Kyu HH , Gakidou E , et al. Estimates of global, regional, and national incidence, prevalence, and mortality of HIV, 1980–2015: the Global Burden of Disease Study 2015. Lancet HIV. 2016;3(8):e361–e387. 27470028 10.1016/S2352-3018(16)30087-X PMC5056319

[3] HIV/AIDS JUNPo . The path that ends AIDS: UNAIDS Global AIDS Update 2023. Geneva; 2023.

[4] Joint United Nations Programme on HIV/AIDS (UNAIDS) . UNAIDS DATA 2022. Geneva, Switzerland: UNAIDS Joint United Nations Programme on HIV/AIDS; 2022. 12349391

[5] International Association of Providers of AIDS Care . Recommendations for the rapid expansion of HIV self‐testing in fast‐track cities. International Association of Providers of AIDS Care; 2017.

[6] World Health Organisation . WHO and partners urge countries to fast‐track implementation and scale‐up of HIV self‐testing and other innovative HIV testing approaches in Asia and the Pacific. Geneva, Switzerland: World Health Organization; 2021.

[7] World Health Organization . WHO recommends HIV self‐testing—evidence update and considerations for success. Geneva, Switzerland: World Health Organization; 2019.

[8] World Health Organisation . HIVST strategic framework—a guide for planning, introducing and scaling up. Geneva, Switzerland: World Health Organization; 2018.

[9] Cassell MM , Girault P , Nith S , Rang C , Sokhan S , Tuot S , et al. A cross‐sectional assessment of HIV self‐testing preferences and uptake among key populations in Phnom Penh, Cambodia. Glob Health Sci Pract. 2022;10(3):e2100412. 36332061 10.9745/GHSP-D-21-00412 PMC9242604

[10] Girault P , Misa Wong C , Jittjang S , Fongkaew K , Cassell MM , Lertpiriyasuwat C , et al. Uptake of oral fluid‐based HIV self‐testing among men who have sex with men and transgender women in Thailand. PLoS One. 2021;16(8):e0256094. 34398926 10.1371/journal.pone.0256094 PMC8367007

[11] Ngoc BV , Majam M , Green K , Tran T , Hung MT , Que AL , et al. Acceptability, feasibility, and accuracy of blood‐based HIV self‐testing: a cross‐sectional study in Ho Chi Minh City, Vietnam. PLoS Glob Public Health. 2023;3(2):e0001438. 36962976 10.1371/journal.pgph.0001438 PMC10022389

[12] Rao A , Patil S , Aheibam S , Kshirsagar P , Hemade P , Panda S . Acceptability of HIV oral self‐test among men having sex with men and transgender population: a qualitative investigation from Pune, India. Infect Dis (Auckl). 2020;13:1178633720962809. 10.1177/1178633720962809 PMC7557648 33110347

[13] Wirtz AL , Naing S , Mon SHH , Paing AZ , Mon EK , Thu KH , et al. High acceptability of HIV self‐testing in a randomized trial among transgender women and men who have sex with men, Myanmar. AIDS Care. 2022;34(4):421–429. 34802339 10.1080/09540121.2021.2005763 PMC8976720

[14] Widyanthini DN , Januraga PP , Wisaksana R , Subronto YW , Sukmaningrum E , Kusmayanti NA , et al. HIV self‐testing for men who have sex with men: an implementation trial in Indonesia. AIDS Care. 2022;34(4):527–534. 33550846 10.1080/09540121.2021.1883509

[15] Rawat S , Solomon SS , Bharat S , Dange A , Srikrishnan AK , Chakrapani V , et al. Community perspectives on making HIV self‐testing accessible as a comprehensive prevention package. National AIDS Control Programme; 2019.

[16] STAR India Consortium . A summary of interventions, lessons learnt, and key takeaways of STAR HIV self‐testing project in India. New Delhi: PATH; 2022.

[17] Wirtz AL , Naing S , Clouse E , Thu KH , Mon SHH , Tun ZM , et al. The Parasol Protocol: an implementation science study of HIV continuum of care interventions for gay men and transgender women in Burma/Myanmar. JMIR Res Protoc. 2017;6(5):e90. 28526661 10.2196/resprot.7642 PMC5451637

[18] Trabwongwitaya P , Songtaweesin WN , Paiboon N , Wongharn P , Moonwong J , Phiphatkhunarnon P , et al. Preference and ability to perform blood‐versus oral‐fluid‐based HIV self‐testing in adolescents and young adults in Bangkok. Int J STD AIDS. 2022;33(5):492–498. 35257618 10.1177/09564624221076955

[19] Tan YR , Kaur N , Ye AJ , Zhang Y , Lim JXZ , Tan RKJ , et al. Perceptions of an HIV self‐testing intervention and its potential role in addressing the barriers to HIV testing among at‐risk heterosexual men: a qualitative analysis. Sex Transm Infect. 2021;97(7):514–520. 33452131 10.1136/sextrans-2020-054773 PMC8543206

[20] Figueroa C , Johnson C , Ford N , Sands A , Dalal S , Meurant R , et al. Reliability of HIV rapid diagnostic tests for self‐testing compared with testing by health‐care workers: a systematic review and meta‐analysis. Lancet HIV. 2018;5(6):e277–e290. 29703707 10.1016/S2352-3018(18)30044-4 PMC5986793

[21] Phongphiew P , Songtaweesin WN , Paiboon N , Phiphatkhunarnon P , Srimuan P , Sowaprux T , et al. Acceptability of blood‐based HIV self‐testing among adolescents aged 15–19 years at risk of HIV acquisition in Bangkok. Int J STD AIDS. 2021;32(10):927–932. 33890847 10.1177/09564624211003742

[22] Shafik N , Deeb S , Srithanaviboonchai K , Ayood P , Malasao R , Siviroj P , et al. Awareness and attitudes toward HIV self‐testing in Northern Thailand. Int J Environ Res Public Health. 2021;18(3):852. 10.3390/ijerph18030852 PMC7908521 33498211

[23] FHI 360 . Report of exploring the uptake and acceptability of HIV self‐testing for men who have sex with men, male sex workers, and transgender people in Nepal. Nepal; 2018.

[24] Shrestha R , Alias H , Wong LP , Altice FL , Lim SH . Using individual stated‐preferences to optimize HIV self‐testing service delivery among men who have sex with men (MSM) in Malaysia: results from a conjoint‐based analysis. BMC Public Health. 2020;20(1):1777. 33238941 10.1186/s12889-020-09832-w PMC7687720

[25] World Health Organization . Differentiated and simplified pre‐exposure prophylaxis for HIV prevention: update to WHO implementation guidance. Geneva, Switzerland: World Health Organization; 2022.

[26] Wirtz AL , Clouse E , Veronese V , Thu KH , Naing S , Baral SD , et al. New HIV testing technologies in the context of a concentrated epidemic and evolving HIV prevention: qualitative research on HIV self‐testing among men who have sex with men and transgender women in Yangon, Myanmar. J Int AIDS Soc. 2017;20(1):21796. 28453242 10.7448/IAS.20.01.21796 PMC5515059

[27] Huang Y‐F , Huang Y‐C , Lo Y‐C , Latkin C , Huang H‐Y , Lee C‐C , et al. Towards the first 90: impact of the national HIV self‐test program on case finding and factors associated with linkage to confirmatory diagnosis in Taiwan. J Int AIDS Soc. 2022;25(3):e25897. 35324087 10.1002/jia2.25897 PMC8944217

[28] Joint United Nations Programme on HIV/AIDS (UNAIDS) . In Danger: UNAIDS Global AIDS Update 2022. Geneva, Switzerland: Joint United Nations Programme on HIV/AIDS (UNAIDS); 2022.

